# Activation of the tail of the ventral tegmental area in response to pup predicting cues in maternal rats

**DOI:** 10.1007/s00429-025-02987-5

**Published:** 2025-07-24

**Authors:** Clara Pérez-Gozalbo, Julia L. Gutiérrez-Arroyo, Manuela Barneo-Muñoz, Fernando Martínez-García, María José Sánchez-Catalán

**Affiliations:** Research Unit on Functional Neuroanatomy (NeuroFun)–Predepartamental Unit of Medicine, Faculty of Health Sciences, Universitat Jaume I de Castellón (UJI), Campus Riu Sec. Av. Vicente Sos Baynat s/n, 12071 Castellón de la Plana, Spain

**Keywords:** Tail of the ventral tegmental area (tVTA), Rostromedial tegmental nucleus (RMTg), Maternal motivation, Reward prediction error, Dopamine, Rat

## Abstract

Motherhood entails brain and behavioral changes associated with increased motivation for pups, ensuring their correct development and survival. Dopamine systems play a crucial role in motivated behaviors, although the exact neurobiological mechanisms underlying maternal behavior remain unknown. The tail of the ventral tegmental area (tVTA) or rostromedial tegmental nucleus (RMTg) is a control center of dopamine systems involved in avoidance and prediction error, among other brain processes. In the present study, we explored its possible contribution in maternal motivation in rats. To do so, we analyzed maternal behavior, as well as the expression of cFos in several brain regions (tVTA/RMTg, anterior–posterior VTA, shell-core ACb, mPFC, LHb, MePD, MPO) of virgin and dam rats in response to pups (Virgin-P, Dam-P) or to pup-predicting cues (absence of pups) (Virgin-NP, Dam-NP). Overall, our results reveal that maternal behavior was only displayed by dams, whereas virgins did not display maternal sensitization in our experimental conditions. Regarding the brain activity, we show that pup-predicting cues induce higher cFos in the tVTA/RMTg of pup-deprived dams compared to non-pup deprived dams and to virgin females, suggesting a role of the tVTA/RMTg in maternal reward prediction error. By contrast, pup exposure or deprivation elicit slight differences on the recruitment of other dopamine and social-related brain regions in our females. Finally, the correlation analysis of activity of brain regions mainly highlights positive correlations in pup-exposed females and scarce correlations in pup-deprived females. Overall, our results reveal a main role of the tVTA/RMTg in maternal reward prediction error.

## Introduction

Maternal behavior includes a set of social behaviors of the female directed to care and promote survival of the offspring until maturation. Impaired maternal behavior affects correct cognitive and emotional development of the offspring (Mehta et al. 2023) and so that ensuring appropriate maternal care is key to promote mental health of future generations (Kundakovic and Champagne 2015). Therefore, it remains essential to understand the neurobiological mechanisms leading to maternal behavior, as well as the factors inducing a lack of motivation to care and protect the offspring.

Fully motivated maternal behavior is induced by a combination of hormonal factors such as sexual steroids and prolactin (Bridges 2020), acting on female’s brain during pregnancy and lactation. Moreover, pup stimuli alone can also induce the expression of maternal behavior, in the absence of hormones (McRae et al. 2023), as for the maternal sensitization observed in virgin rats (Fleming and Rosenblatt 1974) or the quasi-spontaneous maternal care exhibited by virgin female mice (Martín-Sánchez et al. 2015). These females show, however, reduced motivation for pups as compared to lactating dams (Salais-López et al. 2021).

The neurobiological basis of the increased motivation for pups in postpartum females remains partially unclear, although it is generally accepted that the medial preoptic nucleus (MPO) is a central key brain region for maternal behavior. It has been proposed that hormones acting at this level would change the activity of the tegmento-striatal pathway in response to pups, through descending MPO projections to the ventral tegmental area (VTA) (Numan and Young 2016; Fang et al. 2018). However, other brain regions of the reward brain circuitry (e.g. basolateral amygdala) show changes in the response to pups already during late pregnancy (Navarro-Moreno et al. 2022). Thus, it suggests that hormones may act at several levels to induce global changes in the functioning of the motivation and sociosexual brain networks, leading to increased motivation for pups (Navarro-Moreno et al. 2022).

Regarding the brain reward circuit, there is wide agreement on the central role of the tegmento-striatal dopamine pathway, connecting the VTA with the ventral striatum and prefrontal cortex, in the brain processing of goal-directed behaviors (motivated ones), including those social reward-related (Ikemoto 2010; Dölen et al. 2013; Sanchez-Catalan et al. 2014). The VTA is a heterogeneous brain region with its dopamine cells mainly regulated by several neural inputs (Fields et al. 2007; Lammel et al. 2014; Sanchez-Catalan et al. 2014). Among them, the tail of the ventral tegmental area (tVTA) or rostromedial tegmental nucleus (RMTg) is a GABA cell population exerting a major inhibitory control over midbrain dopamine neurons (Sanchez-Catalan et al. 2014). This brain region receives dense inputs from the lateral habenula (Kaufling et al. 2009; Balcita-Pedicino et al. 2011), which is involved in processing aversive information and reward prediction error (Matsumoto and Hikosaka 2009). Therefore, the tVTA/RMTg has been related to aversion and avoidance (Jhou et al. 2009a; Stamatakis and Stuber 2012; Lammel et al. 2012; Sánchez-Catalán et al. 2017; Vento et al. 2017; Proulx et al. 2018; Glover et al. 2023), reward prediction error (Hong et al. 2011; Sánchez-Catalán and Barrot 2022), anxiety and depression (Elmer et al. 2019; Sun et al. 2020; Li et al. 2023a; Wu et al. 2024), motor control (Bourdy et al. 2014; Faivre et al. 2020) and pain (Markovic et al. 2021). Likewise, the role of this brain region in the affective state of drugs of abuse has been widely explored (Jhou et al. 2013; Matsui et al. 2014; Huff and Lalumiere 2015; Fu et al. 2016; Esposito-Zapero et al. 2023). However, there are few studies exploring the role of the tVTA/RMTg in natural reward processing, such as food (Melse et al. 2016; Sánchez-Catalán and Barrot 2022; Schoukroun et al. 2024) or social interaction (Li et al. 2023b). Specifically, the involvement of the tVTA/RMTg in social reward remains poorly understood, and there is nothing known about its possible implication in maternal reward.

Thus, in the present work, we study the behavior of virgin and lactating female rats following pup reintroduction (pup as a reward) or exposure to pup-predicting cues after a period of pup-female separation. After this, we analyze the cFos expression, a proxy of cell activation (Sagar et al. 1988; Chaudhuri 1997), in the tVTA/RMTg and related brain regions, such as mesolimbic (VTAa, VTAp, ACbC, ACbSh) and cortical (mPFC) brain regions, main afferent centers to tVTA/RMTg (LHb) and key brain regions in maternal and social behavior (MPO and MePD). Finally, we also performed a correlation analysis of brain activity (density of cFos immunoreactive cells in the analyzed brain regions) to explore the functioning of the whole brain circuitry.

## Materials and methods

### Animals

Experimental animals were 20 adult female Sprague–Dawley rats of 10–12 weeks of age (Janvier, France). Half of them were pregnant at the arrival to the animal facilities (E15-E16) and half virgins, housed in pairs, one pregnant with one virgin, and under standard conditions (22 ± 1 °C, 12-h light–dark cycle, lights on at 8 am), with *ad libitum* access to water and standard rat chow. To differentiate females, pregnant females were marked with henna on the back. Females were habituated to the facilities for at least one week and daily handled preceding experimentation. Following delivery, litters were not homogenized for postpartum females (7–10 pups per female). Behavioral procedures were conducted between P4 to P9. All procedures were approved by the Committee of Ethics and Animal Welfare of the Universitat Jaume I and Conselleria de Agricultura de la Generalitat Valenciana (Spain) (2020/VSC/PEA/0079) and agreed with directive 86/609/EEC of the European Community on the protection of animals used for experimental and other scientific purposes.

### Experimental design

**Fig. 1:**
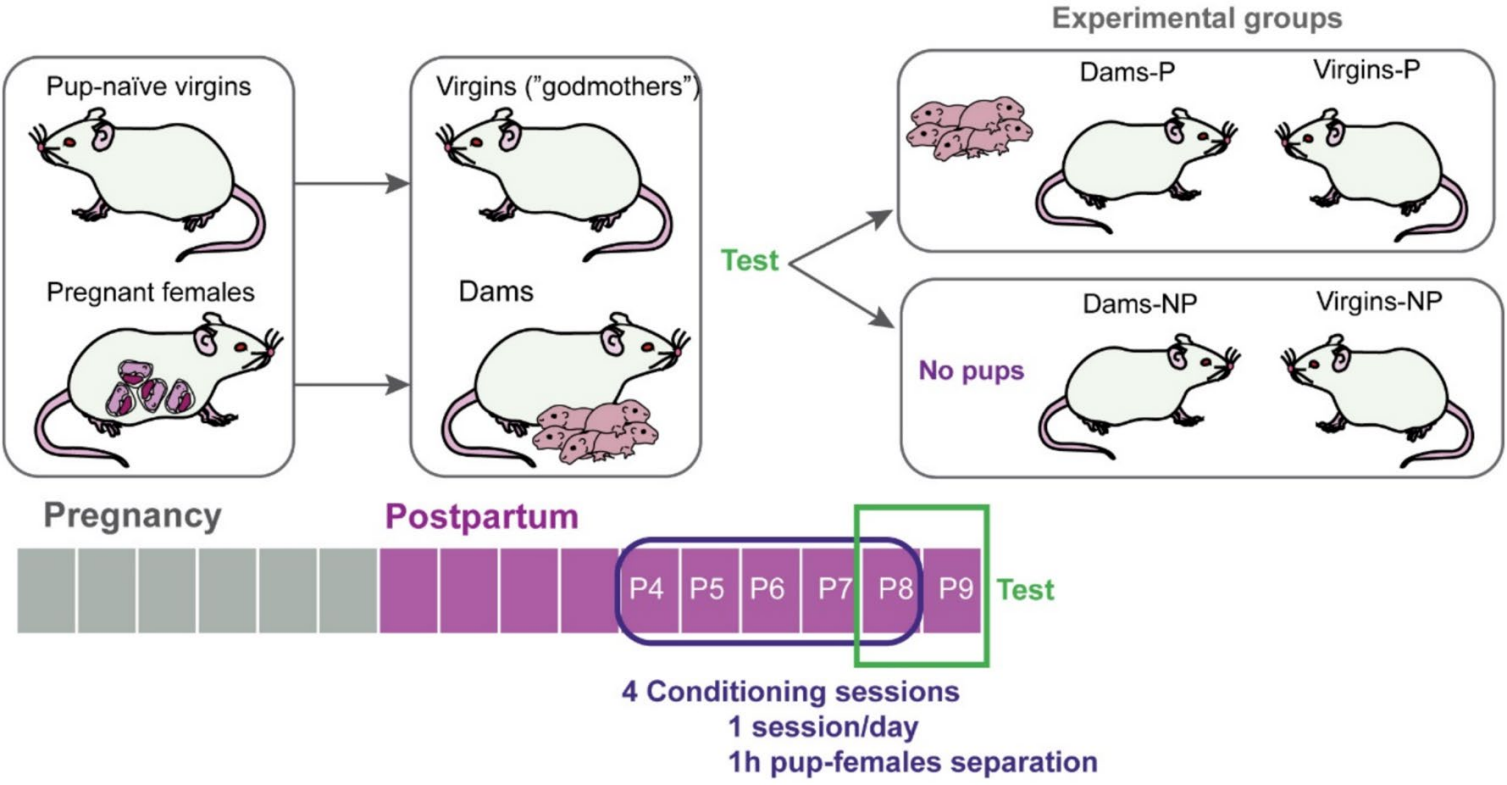
Experimental design. Females were housed in pairs before parturition and underwent 4 conditioning sessions (P4-P7 or P5-P8, pup-females 1 h separation). On the fifth day, the test was performed (P8 or P9) and half of the females received their pups, whereas the other half do not receive them. Behavior of the females was video recorded for 30 min during the conditioning and test sessions by using a video camera. Experimental groups were dams exposed to pups (Dam-P), dams non-exposed to pups (Dam-NP), virgins exposed to pups (Virgin-P), and virgins non-exposed to pups (Virgin-NP).

Females housed in pairs (dam and virgin) underwent the habituation/conditioning phase following 4–5 days after parturition (P4-P5), to 7–8 days postpartum (P7-P8) (4 sessions, 1 session/day). Females underwent one-hour separation from their pups (7–10 pups per litter), previous to the experimental sessions, as previously described (Bayerl et al. 2014), and pups were kept in a separate cage and room and warmed with a thermo pad (36º C). Following separation, females were placed in the experimental room, and pups were reintroduced and spread into the home cage (Fig. 1). On the fifth day, the test was performed (P8-P9), in which, following separation, only half of the females received their pups, whereas the other half do not receive them. Behavior of the females was video recorded for 30 min during the conditioning and test sessions by using a video camera. Following 1 h 30 min of the beginning of the test session, females were deeply anaesthetized and perfused, as described below. Then, the experimental groups were dams exposed to pups (Dam-P), dams non-exposed to pups (Dam-NP), virgins exposed to pups (Virgin-P), and virgins non-exposed to pups (Virgin-NP) (Litter size per group: exposed to pups = 8.2 ± 0.8 pups, non-exposed to pups = 8.8 ± 0.73 pups, comparison between groups: t-test, p = 0.6)

### Behavioral analysis

Maternal behavior of the female rats was analyzed for the conditioning and test sessions for the pup-exposed females, during the first 15 min after pup’s reintroduction. Behavioral analysis was performed as previously described (Navarro-Moreno et al. 2020). For every 5 s, we registered one event of maternal behavior exhibited by the female, according to the following hierarchy (scored from highly to poorly maternal):

- In nest, females stayed inside the nest in close contact with pups.
- Nest Building, the females dug the flake bedding in order to create a hole close to nest.
- On nest, females were located on the nest, near the pups but not in contact with them.
- Approach to pups, olfactory exploration of pups out of the nest, not followed by retrieval.
- Off nest, females were out of the nest and show no interaction with pups.

Then, 180 behavioral events were registered in each animal, distributed among the items described above. Moreover, we assessed the pup retrieval for each female pair, considering the female (dam or virgin) performing retrieval to the nest and time.

Finally, we explored the locomotor activity of the females on the conditioning and test sessions (the first 15 min), by measuring the distance travelled by the females in the home cage (45 × 24 × 20 cm). To do so, we counted the number of crossings in the home cage performed by each female (crossing completed or crossing halfway).

Behavioral assessment was manually scored by the free open-source software BORIS (Behavioral Observation Research Interactive Software, http://www.boris.unito.it) (Friard and Gamba 2016).

### Histological and immunohistochemical procedures

Female rats were anaesthetized with sodium pentobarbital overdose (200 mg/kg, intraperitoneal) (Vetoquinol, Spain) and intracardiac perfused with 100 ml of saline (0.9% sodium chloride), followed by 500 ml of fixative solution (4% paraformaldehyde in 0.1 M of phosphate buffer (PB), pH = 7.4). Brains were removed from the skull, postfixed in the fixative solution overnight at 4ºC, and cryoprotected in 30% sucrose in PB 0.1 M. Coronal brain sections (40 μm) were cut using a freezing slide microtome (Microm HM-450, Walldorf, Germany). For each brain, 4 series of sections were obtained and collected in cryoprotection solution. One series of free-floating sections were used for cFos immunohistochemical analysis. Immunohistochemistry was performed as previously described (Sánchez-Catalán et al. 2017; Navarro-Moreno et al. 2020). Sections were (a) rinsed 3 × 10 min with tris buffer saline (TBS); (b) immersed in 1% H_2_O_2_ and 0.3% Triton X-100 in TBS solution for 30 min for endogenous peroxidase inhibition; (c) rinsed 3 × 10 min in TBS, (d) immersed in a blocking media containing 3% normal goat serum (NGS) and 3% bovine serum albumin (BSA) in TBS 0.01 M pH 8 with 0.3% Triton X-100 for 1 h at room temperature; (e) incubated overnight at room temperature with the primary antibody (guinea pig cFos, Synaptic Systems) diluted 1:5000 in blocking solution; (f) rinsed 3 × 10 min in TBS; (g) incubated in 1:200 dilution of biotinylated rabbit anti-guinea pig secondary antibody (Vector) in blocking solution for 2 h; (h) rinsed 3 × 10 min in TBS; (i) transferred to 1:50 avidin–biotin-peroxidase complex (ABC) (Vectastain-Elite, Vector Laboratories) for 1h30 min at room temperature in TBS; (j) rinsed 2 × 10 min in TBS and 2 × 10 minin tris buffer (TB); (k) the peroxidase activity was revealed with diaminobenzidine tetrahydrochloride (DAB) reaction (0.025% DAB (Sigma-Aldrich), and 0.01% H_2_O_2_ dissolved in TB) for 25 min. The colorimetric reaction was stopped by successive rinsing of sections in TB. Sections were mounted on slides and air-dried overnight. Finally, sections were re-hydrated, cleared with graded ethanol and xylene, and cover slipped in DPX (Scharlau Laboratory).

### Image acquisition and analysis

For evaluation of cFos expression, images of several brain regions (tVTA/RMTg, mPFC, LHb, ACbSh, ACbC, VTAa, VTAp, MPO MePD) in both hemispheres were acquired using a digital camera (DFC495) attached to a Leica DM750 microscope (Leica, AG, Germany). For each region an appropriate magnification was used to cover the region of interest and, when needed, a specific portion of the picture was manually selected as a region of interest (ROI) (Paxinos G 2007) and cFos-ir cells measured with ImageJ software (NIH, Bethesda, MD, USA). Analysis was performed by a person who was blind to the experimental conditions of the samples.

To delineate the boundaries of the tVTA/RMTg and area calculation by ImageJ software with previous neuroanatomical description was used (Sanchez-Catalan et al. 2014)(Paxinos G 2007). The tVTA/RMTg cFos immunoreactive cells (tVTA-ir cells) were manually counted into the ROI (cell counter tool, ImageJ) in all tVTA/RMTg sections of one series (-6 mm to -7 mm from bregma). Then, for each animal, cFos density was calculated by dividing the total number of cFos immunoreactivity (cFos-ir) cells counted in all the sections in both hemispheres by the total sum of the areas of these sections (cFos-ir cells/mm^2^). To analyze VTAa, VTAp, ACbC, ACbSh, mPFC, LHb and MePD and to reduce variability, for each one of these brain centers a specific anteroposterior, dorsoventral and medio-lateral region of interest was selected using the rat brain atlas (Paxinos G 2007); see Figs. 4 and 5) (From bregma: VTAa, -4.8 mm; VTAp-5.52 mm; ACbC and ACbSh, − 1.92 mm; mPFC, 2.76 mm; LHb, − 3.72 mm; MePD, − 3.12 mm). To analyze MPO, three specific regions of interest in three different sections between − 0.24 mm and − 0.84 mm from bregma were analyzed, due to the heterogeneity of this brain region (Simerly 2015)(Paxinos G 2007); see Fig. 5). To quantify the density of cFos-immunoreactive cells in each of these regions, image processing and analysis were conducted as previously described (Navarro-Moreno et al. 2020, 2022).

### Statistical analysis

Data of maternal behavior, locomotor activity, and cFos analysis were first tested for normality (Wilk-Shapiro's test) and homoscedasticity (Levene's test). When applicable, parametric tests were used. The data of maternal behavior in the conditioning sessions was analyzed by Kruskal–Wallis comparison between groups in each conditioning session, whereas in the test session a two-groups comparison of female’s maternal behavior was performed by Mann–Whitney or t-test. Locomotion of females was analyzed by three-way ANOVA with SESSION as intrasubject variable, and FEMALE and STIMULUS as main intersubject factors in the conditioning sessions; and a two-way ANOVA, with FEMALE and STIMULUS as main factors, was performed to analyze locomotion in the test session. When applicable, significant interaction was explored by *post hoc* pairwise comparison with Bonferroni corrections. The data of cFos expression in each brain region was analyzed by two-way ANOVA with FEMALE and STIMULUS as main factors. Bonferroni *post hoc* pairwise comparison was performed when applicable. If normality or homoscedasticity was not accomplished in the four groups, we assessed the differences between main factors separately: FEMALE (all virgins vs dams) and STIMULUS (all pups vs no-pups), by using a two-sample t-test or a Mann–Whitney test. Effect size was assessed by η2 partial, η2, Rosenthal's r or Cohen’s d depending on the applied test. Finally, we performed a Pearson correlation analysis to explore possible differences in brain functioning between the whole brain regions analyzed.

Results were presented as mean ± SEM and data analyzed by using SPSS Statistics for Windows (IBM SPSS Statistics for Windows, Version 29.0). The level of significance was considered at p < 0.05.

## Results

### Maternal behavior and locomotion of dams and virgins

**Fig. 2:**
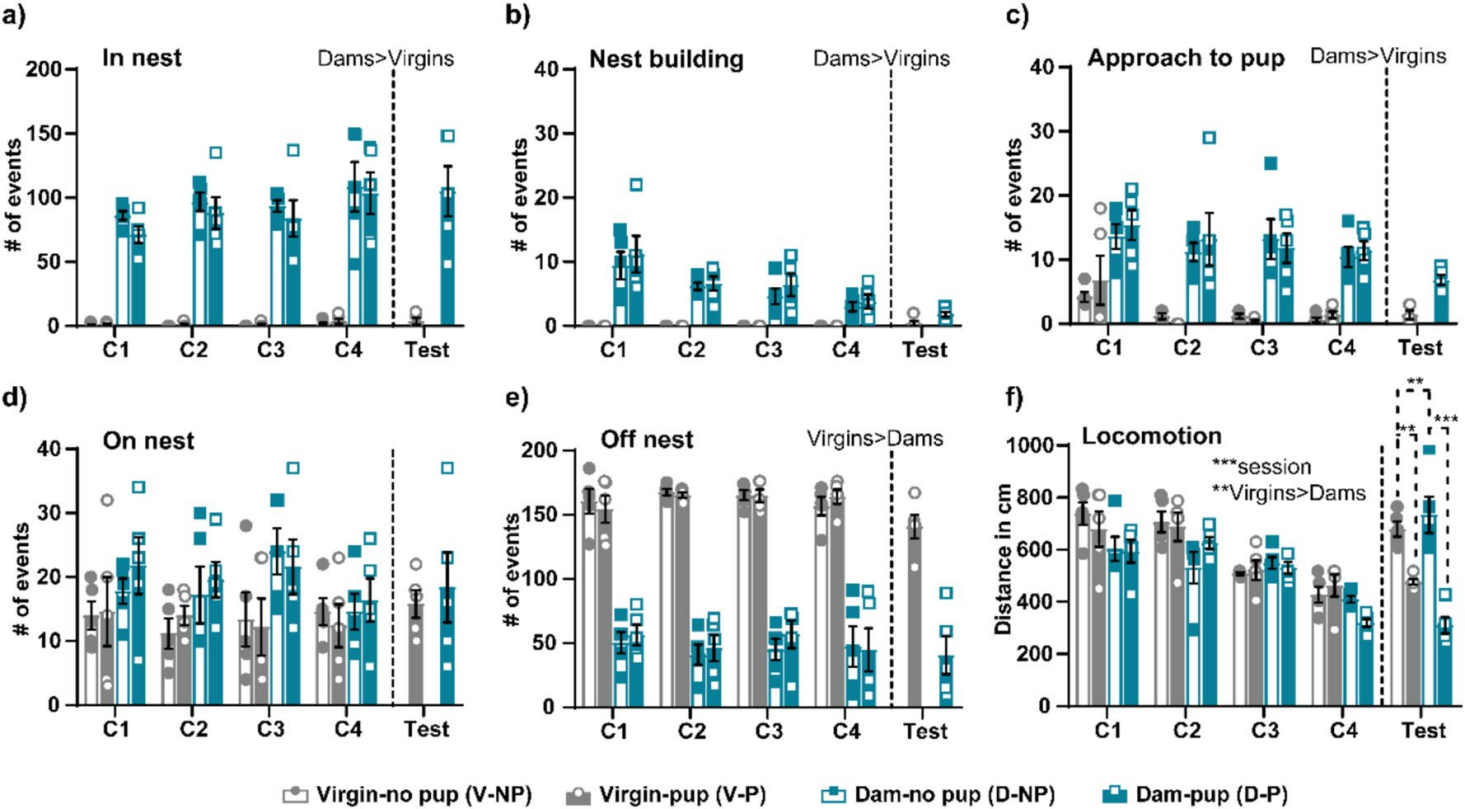
Maternal behavior and locomotion of dams and virgins during the conditioning and test sessions. Following female-pup separation, pups were reintroduced, and behavior of the females was assessed (groups: Dam-P, Virgin-P, Dam-NP. Virgin-NP). In the test session, pups were reintroduced only in half of the groups (Dam-P and Virgin-P). We scored several maternal behavioral events (180 events/15 min) displayed by the females: **(a)** in nest, **(b)** nest building, **(c)** approach to pup, **(d)** on nest and **(e)** off nest. Moreover, we explored the locomotion of the females (**f**). Results are presented as mean ± SEM. Parametric or non-parametric test were performed according to data, **p < 0.01, ***p < 0.001

Following pup-females separation period during the conditioning and test sessions, pups were reintroduced in the home cage and the behavior of the females was video recorded. We scored several maternal behavioral events displayed by the female rats during the sessions. Statistical analysis by Kruskall-Wallis of the results of “in nest” behavior during the conditioning sessions reveal significantly higher score in dams as compared to virgins (C1 p = 0.001, C2 p = 0.001, C3 p = 0.001, C4 p = 0.002, in all cases η^2^ >  = 0.753), a difference also observed between Dams-P and Virgins-P during the test session (Mann–Whitney, Z = -2.652, p = 0.008, r = -0.839) (Fig. 2a). Similar results were obtained with “nest building”, both during the conditioning sessions (Kruskal–Wallis, C1 p = 0.001, C2 p = 0.001, C3 p = 0.001, C4 p = 0.001, in all cases η^2^ = 0.835) and during the test (Mann–Whitney, Z = -2.081, p = 0.037, r = -0.658), with higher values in dams compared to virgins (Fig. 2b), as well as for “approach to pup” behavior (conditioning: Kruskal–Wallis, C1 p = 0.031, C2 p = 0.001, C3 p = 0.002, C4 p = 0.002, in all cases η^2^ > 0.368; and test session: t-test, t = 5.276, p = 0.001, d = 3.53) (Fig. 2c). Otherwise, no significant differences were observed for the “on nest” behavior, neither in the conditioning sessions (Kruskal–Wallis, C1 p = 0.358, C2 p = 0.206, C3 p = 0.147, C4 p = 0.817), nor in the test session (t-test, t = 0.646, p = 0.539) (Fig. 2d). Finally, we observed significant differences on “off nest” behavior, being virgin female groups higher than dam groups (conditioning: Kruskal–Wallis, C1 p = 0.002, C2 p = 0.002, C3 p = 0.002, C4 p = 0.002, in all cases η^2^ > 0.708; and test session: t-test, t = -5.015, p = 0.002, d = 3.36) (Fig. 2e). Moreover, we observed that only dams retrieve pups to the nest when they were reintroduced following pup-females separation, whereas virgins do not display that behavior at all. Overall, our results show that maternal behavior is exclusively displayed in female dams, whereas virgin female rats do not show maternal behavior and they do not develop that behavior even in cohabitation with pups.

Moreover, we assessed the locomotor activity of the females in the home cage during the conditioning and test sessions (Fig. 2f). During the conditioning, the three-way ANOVA show significant differences for the SESSION factor (F_(3,48)_ = 34.894, p < 0.001, η2 partial = 0.686) with a gradual decrease in locomotion through the conditioning sessions, and the FEMALE factor (F_(1,16)_ = 11.970, p = 0.003, η2 partial = 0.428) with higher locomotion in virgins compared to dams. However, no significant differences were observed for the STIMULUS (F_(1,16)_ = 0.90, p = 0.768), neither STIMULUSxFEMALE (F_(1,16)_ = 0.001, p = 0.979), TIMExSTIMULUS (F_(3,48)_ = 0.697, p = 0.558) or TIMExFEMALExSTIMULUS interactions (F_(3,48)_ = 1.750, p = 0.169) (Fig. 2f). In the test session, the two-way ANOVA, show significant differences for STIMULUS (F_(1,16)_ = 57.761, p < 0.001, η2 partial = 0.783), displaying higher locomotion the no-pup females, although no significant differences were observed for the FEMALE (F_(1,16)_ = 1.868, p = 0.191). There is a significant STIMULUSxFEMALE interaction (F_(1,16)_ = 7.178, p = 0.016, η2 partial = 0.310). *Post hoc* comparison with Bonferroni reveals higher locomotion in the absence of pups (Dam-NP > Dam-P, p < 0.001, Virgin-NP > Virgin-P, p = 0.003), and higher locomotion in virgins compared to dams when pups were present (Virgin-P > Dam-P, p = 0.011). Overall, our results show a higher motor activity of the virgins compared to dams along the conditioning and test sessions, and a reduced locomotion in dams when pups were present.

### Impact of pup vs pup-predicting cues exposure on cFos expression in the tVTA/RMTg

**Fig. 3:**
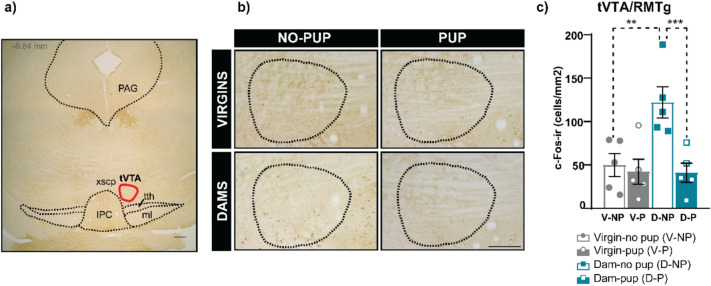
Expression of cFos in the tVTA/RMTg following pup reintroduction or exposure to pup-predicting cues. Following female-pup separation, pups were reintroduced in some groups (Virgin-pup (V-P) and Dam-pup (D-P)), whereas the other groups were simply exposed to the environmental conditions (Virgin-no pup (V-NP) and Dam-no pup (D-NP). **(a)** Example of low-power image of the tVTA/RMTg with one of the antero-posterior levels analyzed (Paxinos G 2007). Red lines indicated the region of interest (ROI) analyzed. Scale bar corresponds to 250 μm. **(b)** Examples of microphotographs of the tVTA/RMTg in the different experimental groups. Scale bar corresponds to 100 μm. (**c)** cFos immunoreactive cells (cFos-ir) in the tVTA/RMTg in the experimental groups. Results are presented as mean ± SEM. Two-way ANOVA with Bonferroni *post hoc* was performed, **p < 0.01, ***p < 0.001. *IPC* interpeduncular nucleus, caudal subnucleus; *ml* medial lemniscus; *PAG* periaqueductal gray; *tth* trigeminothalamic tract; *tVTA* tail of the ventral tegmental area; *xscp* decussation of the superior cerebellar peduncle

In the test session and following pup-females separation, females were exposed to pups or to the environmental conditions associated to pup reintroduction without pups (acting as pup-predicting cues). Our results of cFos expression in the tVTA/RMTg reveal significant differences between experimental groups. Since data accomplished normality and homoscedasticity, we performed a two-way ANOVA with FEMALE (dams vs virgins) and STIMULUS (pups vs no pups) factors. Statistics reveal significant differences for FEMALE (F_(1,16)_ = 6.115, p = 0.025, η2 partial = 0.276) and STIMULUS (F_(1,16)_ = 9.480, p = 0.007, η2 partial = 0.372) factors, as well as the interaction FEMALExSTIMULUS (F_(1,16)_ = 6.483, p = 0.022, η2 partial = 0.288). *Post hoc* pairwise comparison with Bonferroni shows significant differences between Dam-NP vs Dam-P groups (p = 0.001) and between Dam-NP vs Virgin-NP groups (p = 0.003), with higher cFos expression in the Dam-NP group in both cases. Thus, our results show that pup-predicting cues exposure (reward-predicting cues), but no pups, elicits a significant cFos increase in lactating dams, but not in virgins (Fig. 3).

### Impact of pup vs pup-predicting cues exposure on cFos expression in other motivational brain regions and key centers of the sociosexual brain

We explored the cFos expression in several brain regions related to tVTA/RMTg in the different experimental groups to explore the impact of pup reintroduction or exposure to pup-associated cues in their activity.

**Fig. 4:**
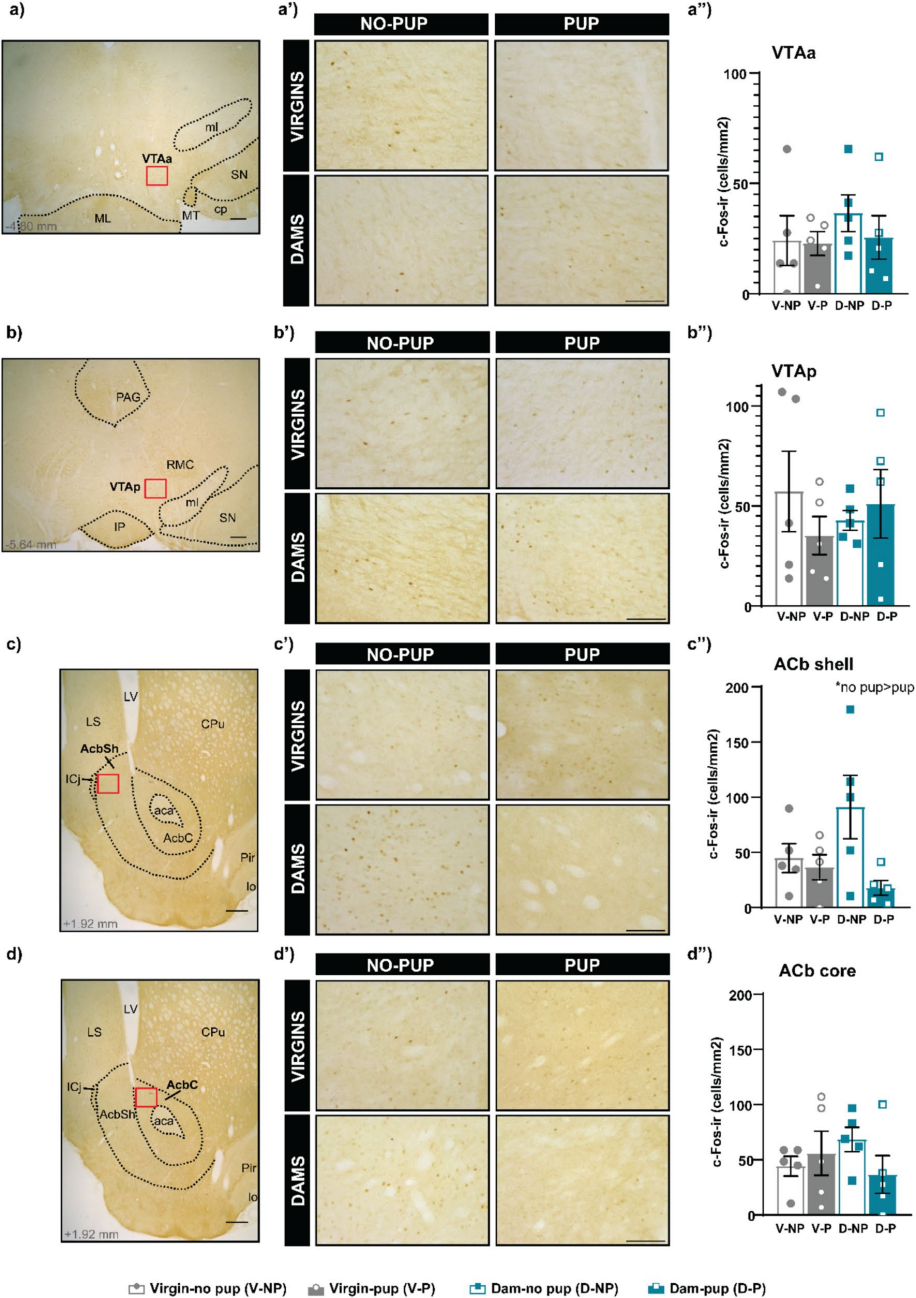
Expression of cFos in dopamine brain regions related to the tVTA/RMTg following pup reintroduction or exposure to pup-predicting cues. Following female-pup separation, pups were reintroduced in some groups ((Virgin-pup (V-NP) and Dam-pup (D-P)), whereas the other groups were simply exposed to the environmental conditions (Virgin-no pup (V-NP) and Dam-no pup (D-NP)). Examples of low-power image of the VTAa (a), VTAp (b), ACbS (c) and ACbC (d) with one of the antero-posterior levels analyzed (Paxinos G 2007). Scale bar corresponds to 250μm. Examples of microphotographs of the VTAa (a’), VTAp (b’), ACbS (c’) and ACbC (d’) in the different experimental groups. Scale bar corresponds to 100μm. cFos immunoreactive cells (cFos-ir) in the VTAa (a’’), VTAp (b’’), ACbS (c’’) and ACbC (d’’) in the experimental groups. Results are presented as mean ± SEM and analyzed by two-way ANOVA, *p<0.05. Statistical analysis of the VTAp corresponds to Log (1+cFos-ir cells/mm2) of the VTAp data. Abbreviations: aca, anterior commissure, anterior part; AcbC, core part of the nucleus accumbens; AcbSh, shell part of the nucleus accumbens; CPu, caudate putamen; ICj, islands of Calleja; IP, interpeduncular nucleus; LS, lateral septum; LV, lateral ventricle; lo, lateral olfactory tract; ml, medial lemniscus; PAG, periaqueductal gray; Pir, piriform cortex; RMC, red nucleus, magnocellular part; SN, substantia nigra; VTAa, anterior ventral tegmental area; VTAp, posterior ventral tegmental area

**Fig. 5:**
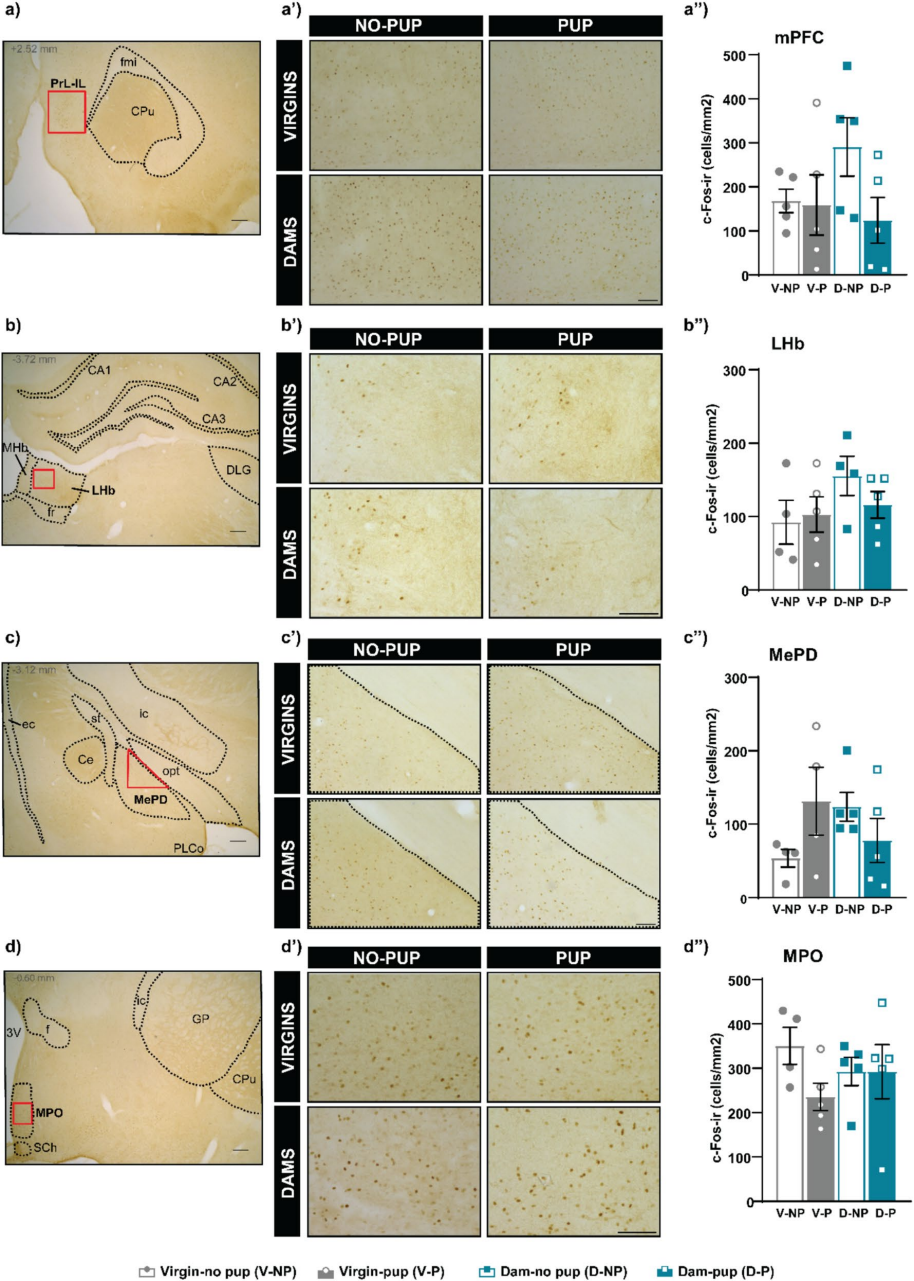
Expression of cFos in other brain regions related to the tVTA/RMTg following pup reintroduction or exposure to pup-predicting cues. Following female-pup separation, pups were reintroduced in some groups (Virgin-pup (V-P) and Dam-pup (D-P)), whereas the other groups were simply exposed to the environmental conditions (Virgin-no pup (V-NP) and Dam-no pup (D-NP)). Examples of low-power image of the mPFC (**a**), LHb (**b**), MPO (**c**) and MePD (**d**) with one of the antero-posterior levels analyzed (Paxinos G 2007). Scale bars correspond to 250 μm. Examples of microphotographs mPFC (a’), LHb (b’), MePD (c’) and MPO (d’) in the different experimental groups. Scale bars correspond to 300 μm in (A’) and (C’) and 100 μm in (B’) and (D’). cFos immunoreactive cells (cFos-ir) in the mPFC (a’’), LHb (b’’), MePD (c’’) and MPO (d’’) in the experimental groups. Results are presented as mean ± SEM and parametric or non-parametric test were used accordingly. Abbreviations: *3 V* 3rd ventricle; *CA1* field CA1 of the hippocampus; *CA2* field CA2 of the hippocampus; *CA3* field CA3 of the hippocampus; *Ce* central amygdaloid nucleus; *CPu* caudate putamen; *DLG* dorsal lateral geniculate nucleus; *ec* external capsule; *f* fornix; *fmi* forceps minor of the corpus callosum; *fr* fasciculus retroflexus; *GP* globus pallidus; *LHb* lateral habenula; ic, internal capsule; *MePD* medial amygdaloid nucleus, posterodorsal part; *MHb* medial habenula; *MPO* medial preoptic nucleus; *opt* optic tract; *PLCo* posterolateral cortical amygdaloid nucleus; *PrL-IL* prelimbic and infralimbic cortex; *SCh* suprachiasmatic nucleus; *st* stria terminalis

Since one of the main outputs of the tVTA/RMTg is the VTA, we explored the brain activity in the anterior and posterior VTA (VTAa, VTAp), as well as their main efferents, the ACb core and shell (ACbC and ACbSh) and mPFC. A two-way ANOVA was performed to analyze the results of VTAa, and no significant differences were found between FEMALE (F_(1,16)_ = 0.715, p = 0.410) or STIMULUS (F_(1,16)_ = 0.479, p = 0.499), neither significant interaction FEMALExSTIMULUS (F_(1,16)_ = 0.290, p = 0.598; Fig. 4a, a’, a’’). Log transformed data on cFos in the VTAp were analyzed using a two-way ANOVA, which rendered no significant differences between factors (FEMALE, F_(1,16)_ = 0.003, p = 0.956; STIMULUS, F_(1,16)_ = 0.598, p = 0.451) neither FEMALExSTIMULUS interaction (F_(1,16)_ = 0.006 p = 0.940; Fig. 4b, b’, b’’). Regarding the ACb, a two-way ANOVA of the ACbSh data reveal significant differences between STIMULUS, F_(1,16)_ = 5.680, p = 0.030, η2 partial = 0.262), with no-pup groups displaying higher cFos levels than pup-exposed ones. By contrast, no differences were found between FEMALE (F_(1,16)_ = 0.623, p = 0.431), neither the interaction FEMALExSTIMULUS (F_(1,16)_ = 3.604, p = 0.076) (Fig. 4c, c’, c’’). Concerning the ACbC and the mPFC, no significant differences were observed (ACbC: FEMALE F_(1,16)_ = 0.026 p = 0.87), STIMULUS F_(1,16)_ = 0.450 p = 0.512, FEMALExSTIMULUS F_(1,16)_ = 2.123 p = 0.164, Fig. 4d, d’, d’’) (mPFC: FEMALE F_(1,16)_ = 0.617 p = 0.444, STIMULUS F_(1,16)_ = 2.477 p = 0.135, FEMALExSTIMULUS F_(1,16)_ = 1.981 p = 0.178, Fig. 5a, a’, a’’).

Besides analyzing the activity of the main efferences of the tVTA/RMTg and the associated ascending dopaminergic pathways, we also explored the main afferent to the tVTA/RMTg, the LHb. Our data on cFos expression in the LHb do not reveal significant differences (FEMALE F_(1,14)_ = 2.416 p = 0.142, STIMULUS F_(1,14)_ = 0.346 p = 0.566, FEMALExSTIMULUS interaction F_(1,14)_ = 1.037 p = 0.326). cFos expression was mainly observed in the medial part of the LHb, compared to the lateral part, although full LHb analysis is displayed in the present work, we performed a restricted analysis of the medial LHb and no differences were observed either (data not shown) (Fig. 5b, b’, b’’).

Finally, we assessed brain activity in the two key centers of the socio-sexual/maternal brain, namely the MePD and MPO. First, main factors were assessed for the data on MePD by t-test (parametric data by factor) and no differences were found: STIMULUS, t = 0.290, p = 0.775, FEMALE, t = -0.263, p = 0.796 (Fig. 5c, c’, c’’). Second, the two-way ANOVA of the MPO activity did not show differences: FEMALE F_(1,15)_ = 0.000 p = 0.993, STIMULUS F_(1,15)_ = 1.739 p = 0.207 and FEMALExSTIMULUS F_(1,15)_ = 1.705 p = 0..211) (Fig. 5d, d’, d’’).

### Correlation analysis between activity of brain regions

Finally, we performed a Pearson correlation analysis of the cFos expression levels between the previous analyzed brain regions to assess brain functioning in virgins and dams exposed to pups or only to pup-predicting cues following a period of pup-females separation.

On one hand, statistics reveal positive significant correlations in virgins exposed to pups (Virgin-P): tVTA/RMTg activity correlates with MPO (p = 0.045) and mPFC (p = 0.005), ACbSh activity correlates with ACbC (p = 0.03) and MePD (p = 0.005), mPFC activity correlates with ACbC (p = 0.038). Moreover, in virgins non-exposed to pups (Virgin-NP), a negative correlation was observed between MPO and mPFC (p = 0.021) in virgins non-exposed to pups.

On the other hand, correlation analysis shows positive significant correlations in dams exposed to pups (Dam-P): tVTA/RMTg activity correlates with VTAp (p = 0.034) and MePD (p = 0.012), ACbSh activity correlates with ACbC (p = 0.01) and VTAa (p = 0.003), ACbC activity correlates with VTAa (p = 0.001). Finally, in dams non-exposed to pups (Dam-NP), a positive correlation of cFos expression was observed between MPO and LHb (p = 0.025) (Table 1).

**Table 1:**
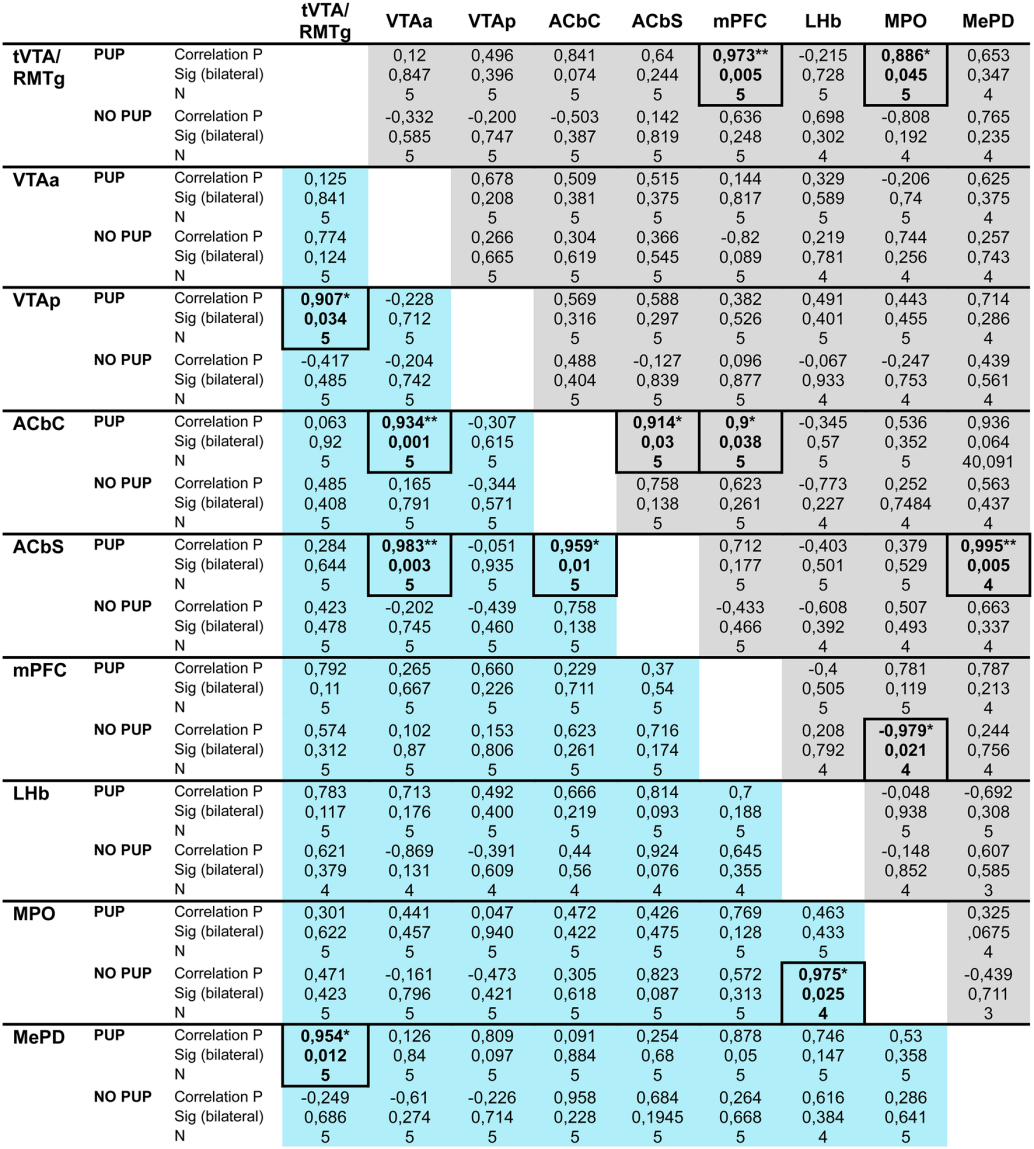
Correlation analysis of cFos expression in different brain regions of virgins and dams

Pearson correlation analysis of cFos levels in the brain regions analyzed previously. Virgins and dams are represented in grey and blue color, respectively. *p < 0.05, **p < 0.01

Overall, multiple positive correlations were found in females, in both, virgins and dams, exposed to pups, whereas a few correlations were found in in pup-deprived females.

## Discussion

In the present study, we assessed the role of the tVTA/RMTg in brain processing of maternal reward in rats, and the activity of other reward and social-related brain regions (anterior and posterior VTA, ACbSh and ACbC, mPFC, LHb, MePD and MPO) following pup exposure or absence, which allows assessing brain processing of pup-derived and pup-predicting cues occurring in a maternal context. To do so, we analyzed maternal behavior, as well as the expression of cFos in several brain regions of virgin and dam rats in response to pups (Virgin-P, Dam-P) or to pup-predicting cues (absence of pups) (Virgin-NP, Dam-NP). Last, we ascertained possible correlations between patterns of brain activity between brain regions. Overall, our results reveal that maternal behavior was only displayed by dams, whereas virgins did not display maternal sensitization in our experimental conditions. Regarding the activity of specific brain regions, we show that pup-predicting cues induce higher cFos in the tVTA/RMTg of pup-deprived dams compared to non-pup deprived dams and to virgin females, suggesting a role of the tVTA/RMTg in maternal reward prediction error. By contrast, pup exposure or deprivation elicit slight differences on the recruitment of other dopamine and social-related brain regions in our experimental females. Finally, the correlation analysis of activity of brain regions mainly highlights positive correlations in pup-exposed females and scarce correlations in pup-deprived females.

### Virgins do not display maternal sensitization

Our behavioral results reveal that only dam rats display maternal behavior during the experimental sessions. Lactating females have higher events in nest, nest building and approach to pup, whereas they display lower events off nest compared to virgins, which is the lowest maternal behavior assessed. Moreover, pup retrieval to the nest following pup reintroduction was exclusively performed by dams. No differences were observed between virgins and dams with regard to “on nest” behavior. After pup reintroduction, dams spent most of the time around the nest taking care of pups, whereas virgins were sometimes close to the nest, probably looking for social interaction.

Our results indicate that virgins were not maternally sensitized despite being cohabitated with pups since birth. They do not display maternal behavior during the conditioning sessions, either on the test session performed 8–9 days postpartum. According to previous literature, virgin rats initially avoid pups (Mayer and Rosenblatt; Fleming et al. 1981) but can be maternally sensitized by exposure to foster young pups for 6–8 days approximately (range of 2–11 days) (Rosenblatt 1967; Fleming and Rosenblatt 1974; Furuta and Bridges 2009). In those previous studies, virgins were exposed 24 h to foster pups renewed each day until maternal behaviors appeared. This behavioral sensitization seems hormone independent since ovariectomized females can also be maternally sensitized (Rosenblatt 1967). Similarly, virgin female mice display maternal care nearly spontaneously (Stolzenberg and Rissman 2011; Martín-Sánchez et al. 2015). Indeed, virgin females cohabitating with a mother and her litter are called “godmothers” since they share caring behavior to the offspring with dams (Martín-Sánchez et al. 2015), although they show relatively reduced maternal motivation, due to the lack of the hormonal influence that affects mothers’ brain (Salais-López et al. 2021). These findings highlight important differences in maternal behavior between rodent species, rats and mice.

By contrast to previous works, our virgin rats show no evidence of maternal sensitization, a fact likely due to the presence of the dam during cohabitation with pups. We wonder if this might be due to maternal aggression, expressed against intruders by mothers in late pregnancy (Bosch 2013), although we do not observe clearly aggressive events from dams to virgins, probably because they were cohoused since their arrival to the animal facilities (pregnancy period), and they were already known animals. Even so, the predominant position of the dam in the nest while taking care of the pups, could prevent the virgin to have access to pup-derived proximal stimuli, that could be key for development of maternal sensitization (Stern 1997; Lévy et al. 2004).

Regarding the locomotor activity of the animals during the conditioning sessions, there was a significant gradual reduction in locomotion during postpartum, likely due to habituation to the experimental conditions, with virgins displaying higher locomotion than dams in the conditioning sessions, which is expected since virgins and dams are mostly “off nest” and “in nest”, respectively. Accordingly, in the groups with pup reintroduction in the test session, higher locomotion was observed in virgins, as compared to dams. Pup-deprived females (both virgins and dams) display the highest locomotion, which could also mirror an exploratory behavior of the females (Bromberg-Martin et al. 2010).

### tVTA/RMTg involvement in maternal reward prediction error

Our analysis of cFos expression in these animals reveal that cFos-ir cells are denser in the tVTA/RMTg of pup-deprived dams (Dams-NP) as compared to non-pup-deprived dams (Dam-P). In our experiment, the conditioning sessions allowed association of the experimental room with the reintroduction of pups, thus ensuring that the environmental cues predict the reintroduction and presence of pups, a cue-reward association similar to the one observed with other natural rewards (Sánchez-Catalán and Barrot 2022). In these experimental conditions, if dams find pup rewarding, they would expect and predict to receive the reward.

By contrast, for virgin females, even after maternal sensitization, pups have a reduced rewarding value as compared to dams (Fleming et al. 1994), as previously shown using different experimental setups (Lee et al. 2000; Mattson et al. 2001). Related with this, our results reveal that cFos expression is significantly higher in the Dam-NP compared to the Virgin-NP group, suggesting that there is a differential reward value of pups between dams and virgins (not sensitized in our experimental conditions) (Matsushita et al. 2015; Salais-López et al. 2021), likely indicating that pups have no reward value for virgins.

Therefore, the increase in cFos expression in the tVTA/RMTg seems related to the pup rewarding properties, and specifically when pups are expected but not reintroduced. The associative learning between pups and experimental cues leads to reward-associated cues adopting specific salience and generate reward prediction error, which is the difference between the real reward and the expected one, and allows adjusting learning or correcting behavior to optimize response (Chang et al. 2015). Both salience and reward prediction error, can take place in midbrain dopamine neurons (Bromberg-Martin et al. 2010; Berridge 2012; Schultz 2017), whose activity is regulated by several excitatory and inhibitory inputs (Watabe-Uchida et al. 2012). Among them, the tVTA/RMTg is a key inhibitory input to dopamine cells.

Our results agree, therefore, with previous studies showing that the tVTA/RMTg encodes reward prediction error (Matsumoto and Hikosaka 2007; Hong et al. 2011; Sánchez-Catalán and Barrot 2022). When error is detected (pup reward is predicted but nor present), tVTA/RMTg cells are activated and cFos is highly expressed. This has also been observed in punishment prediction processes, as repeated footshocks (repetition of discrete emotional events enabling to code prediction error) or shock-predictive cues exposure (Jhou et al. 2009a; Brown and Shepard 2013; Sánchez-Catalán et al. 2017; Li et al. 2019b). These studies and our results highlight that neutral environmental cues can be associated with obtaining reward or punishment, thus, adopting specific salience and value (Bromberg-Martin et al. 2010), encoded in the tVTA/RMTg cells activity. Likewise, it has been shown that GABA cells of the VTA inhibit dopamine neurons when reward is expected, contributing to prediction error (Eshel et al. 2015). The GABA cells of the tVTA/RMTg constitute a heterogeneous population, including cells responding to reward-associated cues, with either excitation or inhibition responses to those cues (Li et al. 2019a). The tVTA/RMTg cells activated in our experimental animals may likely be the ones corresponding to the subpopulation of reward cue-excited GABA neurons of the tVTA/RMTg.

Overall, our tVTA/RMTg results suggest a role of this brain region in maternal reward prediction error, hence, highlighting that this brain region must be considered as a relevant player encoding reward prediction error (Watabe-Uchida et al. 2017).

### Effect of pup exposure or deprivation on the recruitment of reward and social-related brain regions

By using cFos expression, we also assessed the activity of other reward and social brain-related regions to elucidate if pup exposure or deprivation may have an effect depending on the hormonal status of the female in a maternal context. Additionally, we performed a correlation analysis of activity between brain regions of our experimental females.

Surprisingly, the analysis of the VTA (anterior and posterior) reveal no significant differences between experimental females. Midbrain dopamine neurons regulate reward-related learning in positive and negative direction (Chang et al. 2015) by encoding reward prediction error (Bromberg-Martin et al. 2010; Schultz 2017). Indeed, it has been shown that VTA dopamine cells can code reward prediction error in maternal care behavior (Xie et al. 2023). But also, their activity is mainly controlled by the tVTA/RMTg, where activity differences were observed in our experimental females. The lack of VTA differences in our experimental conditions may be due to several factors: a) the activity of dopamine neurons code reward size (Eshel et al. 2016), but litters were not homogenized in our experiment, which could increase variability between females, thus, masking possible differences; b) we analyzed specific and representative frameworks of the VTA, but we did not differentiate the type of neurons being activated within the VTAa or VTAp; c) cFos approach provides indirect information about neuronal activity and some activity changes can be unnoticed, since it is not so precise as the electrophysiological approach, which can be time-locked to the actual event.

Besides this lack of differences between experimental groups, positive significant correlations were observed in cFos activity between the VTAa and the ACb (shell and core), which is restricted to dams exposed to pups. These results agree with the tegmento-striatal functioning (Ikemoto 2010) and the exclusive rewarding value of pups for dams (see behavioral results). Likewise, a positive correlation was observed between VTAp and tVTA/RMTg in the Dam-P group. Since the tVTA/RMTg is an inhibitory control center of midbrain dopamine neurons, a negative correlation was expected. However, we did not differentiate the type of neurons activated in the VTA, thus, most of them could be GABA cell interneurons exerting the same effects that some tVTA/RMTg cells (Eshel et al. 2015).

Regarding the ACb, we observed significant differences in the shell with higher activity in the females non-exposed to pups compared to those exposed to pups, mainly due to Dam-NP group. By contrast, no differences in cFos expression were observed in the ACbC. Dopamine release and activity in ACb subregions, shell and core, seems to be specifically tuned to support plasticity in learning and motivational contexts (Cacciapaglia et al. 2012; Badrinarayan et al. 2012). Activity in the ACbSh is likely encoding incentive salience of the stimuli (reward or reward-predicting cue) (Cacciapaglia et al. 2012) and mainly involved in negative reward prediction error (reward is absent or smaller than predicted) (Saddoris et al. 2015), which agrees with our results. Otherwise, activity in the ACbC is related to prediction error, coding the individual value of predicted outcome, and changing dynamics by delay of reinforcement or reward omission to an appropriate decision-making (Sugam et al. 2012; Saddoris et al. 2015). In our experiment, cFos analysis is not suitable to evaluate precise temporal shifts in reward detection, thus, explaining the negative results in the ACbC. In this sense, brain activity correlations reveal some interesting relations. First, positive correlation between ACbC and ACbSh was found in virgins and dams exposed to pups, in agreement with the functional connectivity within the ACb (Zahm 1999). Second, a positive correlation between ACbC and mPFC was found in virgins exposed to pups, both regions involved in goal-directed behavior (Gill et al. 2010), which could mirror the decision-making process of the virgins to approach or not to the pups, especially due to the presence of the dam. Third, a positive correlation between ACbSh and MePD was found in virgins exposed to pups, likely expected due to the amygdalostriatal pathways connecting both centers (Novejarque et al. 2011).

We also explored the activity of the mPFC in our experimental females, although differences were not observed between females or stimuli, a positive correlation was found in cFos expression between the tVTA/RMTg and mPFC in virgins exposed to pups. Both brain regions are indirectly and directly connected (Sanchez-Catalan et al. 2014; Cruz et al. 2021), and its activity can code reward expectancy related to spatial environmental information (Pratt and Mizumori 2001; Jo et al. 2013). Therefore, our finding of correlation in virgins when pups are reintroduced suggests a possible value of pups as predictors for virgins, e.g. due to anticipating a more comfortable or relaxed behavior/attitude of the dam.

Likewise, we did not observe differences in the activity of the LHb of our experimental females, although the highest activity was displayed in the dams non-exposed to pups. The LHb encodes prediction error signals to dopamine system depending on tVTA/RMTg cells (Hong et al. 2011; Brown et al. 2017), thus, we expected a similar effect as in the tVTA/RMTg. Nevertheless, related issues to the technique used and, probably, the small size of the samples, could mask some discrete events occurring in those brain regions. As described above, the cFos expression was mainly observed in the medial part of the LHb, which is the subregion mainly projecting to the rostral part of the tVTA/RMTg and the VTA (Jhou et al. 2009b; Petzel et al. 2017) and receiving from the medial preoptic area (Yetnikoff et al. 2015). Hence, due to those connections, it could be the subregion participating in maternal brain processing.

Finally, we assessed the activity of MePD, a key brain region in chemosensory processing and social reward (Hu et al. 2021), and the MPO, a key brain region in maternal behavior (Numan and Woodside 2010). Indeed, the neural pathway amygdala-hypothalamus is critical for social motivation, since it is able to regulate dopamine release in the ACb and seems to be specific of natural rewards (Hu et al. 2021). In our experimental conditions, no differences in cFos expression were observed in those brain regions between female groups. Contrary to previous works in mice, showing increased activity following pup exposure (Tsuneoka et al. 2013; Navarro-Moreno et al. 2022), we did not observe a specific increase activity in females exposed to pups. Regarding MePD correlation results, a positive correlation between MePD and tVTA/RMTg was only observed in dams exposed to pups. The amygdala and the tVTA/RMTg display scarce no direct neuroanatomical connections (Sanchez-Catalan et al. 2014), although indirect ones cannot be ruled out. Medial amygdala (specifically MePD) express high levels of prolactin and oestrogens receptors (Petrulis 2013; Salais-López et al. 2017) and is essential to integrate sensory information leading to specific social behaviour, such as maternal care (Chen et al. 2019).

Moreover, correlation between the tVTA/RMTg and MPO was observed only in virgins exposed to pups, suggesting a functional connection between both regions (Sanchez-Catalan et al. 2014). By contrast, a negative correlation between mPFC and MPO was displayed in pup-deprived virgin females, likely mirroring the brain codification of decision-making and goal-directed behavior in the absence of pups (Alsina-Llanes and Olazábal 2020). Finally, a positive correlation between LHb and MPO was found in pup-deprived dams, likely suggesting that LHb may serve as prediction node in a maternal brain context, which agrees with previous studies showing the relevance of including LHb into the maternal brain circuitry (Felton et al.; Benedict et al. 2025).

## Conclusions

By using virgin and lactating females, we indirectly assess the relevance of hormonal status in the context of maternal care. Our behavioral results revealed that dam rats display strong maternal behavior after separation from their pups, whereas virgin female rats do not display maternal sensitization besides the continuous exposure to pups along 8–9 days.

By exploring the recruitment of specific transcription factors (cFos), we provide indirect information about neuronal activity and plasticity (despite its poor time resolution). Overall, our present results show an overview of brain processing of pup-derived and pup-predicting cues in the maternal brain, and support the involvement of the tVTA/RMTg in maternal reward prediction error, highlighting for the first time, the role of the tVTA/RMTg in maternal behavior. However, no sharp differences were found in other reward and social-related brain regions in our experimental females, but as previously discussed, fine tuning of activity in specific brain regions can be unnoticed by using indirect assessment of neural inactivation. Furthermore, the correlation activity of reward and social brain regions contributes to understanding the complex neural circuitry regulating maternal care.

## Data Availability

No datasets were generated or analysed during the current study.
